# Novel technique for comminuted patellar fixation using suture tape in spiderweb configuration

**DOI:** 10.3389/fsurg.2024.1377921

**Published:** 2024-08-29

**Authors:** Wei Jie Tee, Kuei Siong Andy Yeo, David Thai Chong Chua, Ing How Moo

**Affiliations:** Department of Orthopaedic Surgery, Changi General Hospital, Singapore, Singapore

**Keywords:** patella—injuries, patella—surgery, fracture fixation without cerclage cables, novel technique, suture tape

## Abstract

**Introduction:**

Patella fractures account for 1% of skeletal fractures in orthopedic surgery. Simple two-part patella fractures are uncommon; most fractures are comminuted with significant articular involvement. Traditionally, patella fractures have been fixed using a tension band technique with cerclage wire, which has several complications including soft-tissue irritation, implant migration, and breakage, leading to secondary implant removal in up to 37% of patients. Newer fixation methods using cannulated screws, hook plates, mesh plates, and locking plates show promise but are costly and require extensive soft-tissue dissection. There is a need for a better alternative, especially for the elderly with osteoporotic bones.

**Recent developments:**

Various authors have described patellar fixation techniques augmented with sutures and suture tape, showing satisfactory outcomes. This paper proposes a novel all-suture tape method for patellar fixation, suitable for common types of patella fractures including AO 34C1.1 (transverse), AO 34C2 (transverse and split), and 34C3 (comminuted). Suture tape is biomechanically superior in both soft tissue and bone.

**Operative technique:**

The patient is laid supine with the knee in full extension. A standard anterior midline approach is adopted. After reducing the fracture fragments and securing them with K-wires, non-absorbable suture tapes are used instead of cerclage wire. The tapes are passed multiple times through the soft tissue, creating loops that are then tensioned to compress the fracture fragments. The technique is completed by creating a tension band fixation with additional suture tapes.

**Expected outcomes:**

This technique offers several benefits, including reduced operative time, minimized soft-tissue dissection, and lower risk of implant prominence and irritation. The suture tape's superior tensile strength and low tissue reactivity reduce complications and the need for secondary surgeries. Early results from two cases show union achieved at 3 months without complications, with patients regaining full range of motion.

**Conclusion:**

This preliminary technical paper demonstrates the feasibility of using non-metallic implants for patella fracture fixation. The proposed method shows promising results, suggesting a potential shift in the approach to fracture fixation. Further research and larger cohort studies are needed to validate these findings.

## Introduction

Patella fracture accounts for 1% of skeletal fractures in orthopedic surgery. Although several subtypes have been described for patella fractures, a simple two-part patella fracture is uncommon and more fractures are comminuted with significant articular involvement.

Patella fracture is traditionally fixed by means of a tension band technique using cerclage wire fixation with wires placed in a figure-of-eight configuration. Additional smaller fragments can be reattached to the main fragment using compression screws. However, this prevalent method is associated with several complications and challenges, such as unnecessary dissection of the surrounding soft tissue, implant migration, prominence, soft-tissue irritation, and implant breakage. This is associated with secondary implant removal in up to 37% of the patients (1–3). This method requires multiple intraoperative imaging to ensure proper implant position and requires the knee to be flexed intraoperatively to pass two parallel K-wires. This may displace the fracture reduction. Manipulation of the metallic wires can be difficult while attempting to place the cerclage wire close to the patella. Further, the various modifications of the traditional technique have not solved the challenges of fixing a severely comminuted fracture.

Recent studies on newer fixation methods using cannulated screws, hook plate (4), mesh plate (5, 6), variable angle, and fixed angle locking plates (7) show they are promising but carry the disadvantage of being costly and requiring extensive soft-tissue dissection. Fixation depends on reliable bone quality, screw purchase, and requires extensive use of intraoperative imaging. Lack of adequate and reliable fixation construct for patella fracture may predispose the patient to early osteoarthritis.

In the elderly population, fixation failure and cut-out rates are known to be even higher due to osteoporotic bones (8). Associated comorbidities may also preclude the surgeon from performing long surgeries and secondary surgeries. With an aging population and increasing prevalence of osteoporosis, bone stock and bone quality to allow for adequate fixation using the traditional technique is commonly compromised. All in all, there is a need to develop a better alternative method of fixation. Recently, various authors have described different patellar fixation techniques augmented with sutures (9, 10) and suture tape (11, 12) with satisfactory outcomes. The use of suture for fixation in other bones has also been described (13) in the literature.

This is a preliminary technical paper to propose a novel all-suture tape method of patellar fixation that is suitable for common types of patella fracture including AO 34C1.1 (transverse), AO 34C2 (transverse and split), and 34C3 (comminuted). We chose a suture tape that is biomechanically superior both in soft tissues and bones (14). Indications for fixation include the following: (1) displaced patellar fractures with disruption of extensor mechanism, (2) incongruity in the articular surface of the patellar of >2 mm, and (3) a gap between fracture fragments of >3 mm. In particular, we feel that the technique is particularly suited to fractures where good bone contact between fragments on the articular aspect of the fracture cannot be achieved. It will overcome the challenges seen in the current fixation methods and may result in reduction in operative time, radiation exposure, and the need for secondary surgery.

## Operative technique

The patient is laid supine on the operating table with a sandbag placed under the ipsilateral buttock to keep the knee in an upright position. A tourniquet is applied to the proximal thigh prior to draping.

A standard anterior midline approach to the patella is adopted. A full thickness fasciocutaneous flap is developed medially and laterally to preserve the vascularity to the skin and to minimize unnecessary soft-tissue dissection. The proximal and distal extent of the incision is made to expose the insertion of the quadriceps tendon into the upper pole and origin of the patella tendon into the lower pole of the patella. Any remnant soft tissues are dissected off the fracture site to avoid soft-tissue interposition while clearing off fracture hematoma to prepare for fixation.

A sterile rolled tower is then placed under the ipsilateral ankle to keep the knee extended. The fracture fragments are reduced to restore the articular congruency. The reduction is then temporarily maintained using K-wires or reduction clamps. The quality of reduction of the articular surface is then checked under direct visualization and confirmed with orthogonal image intensifier imaging.

**Figure 1:**
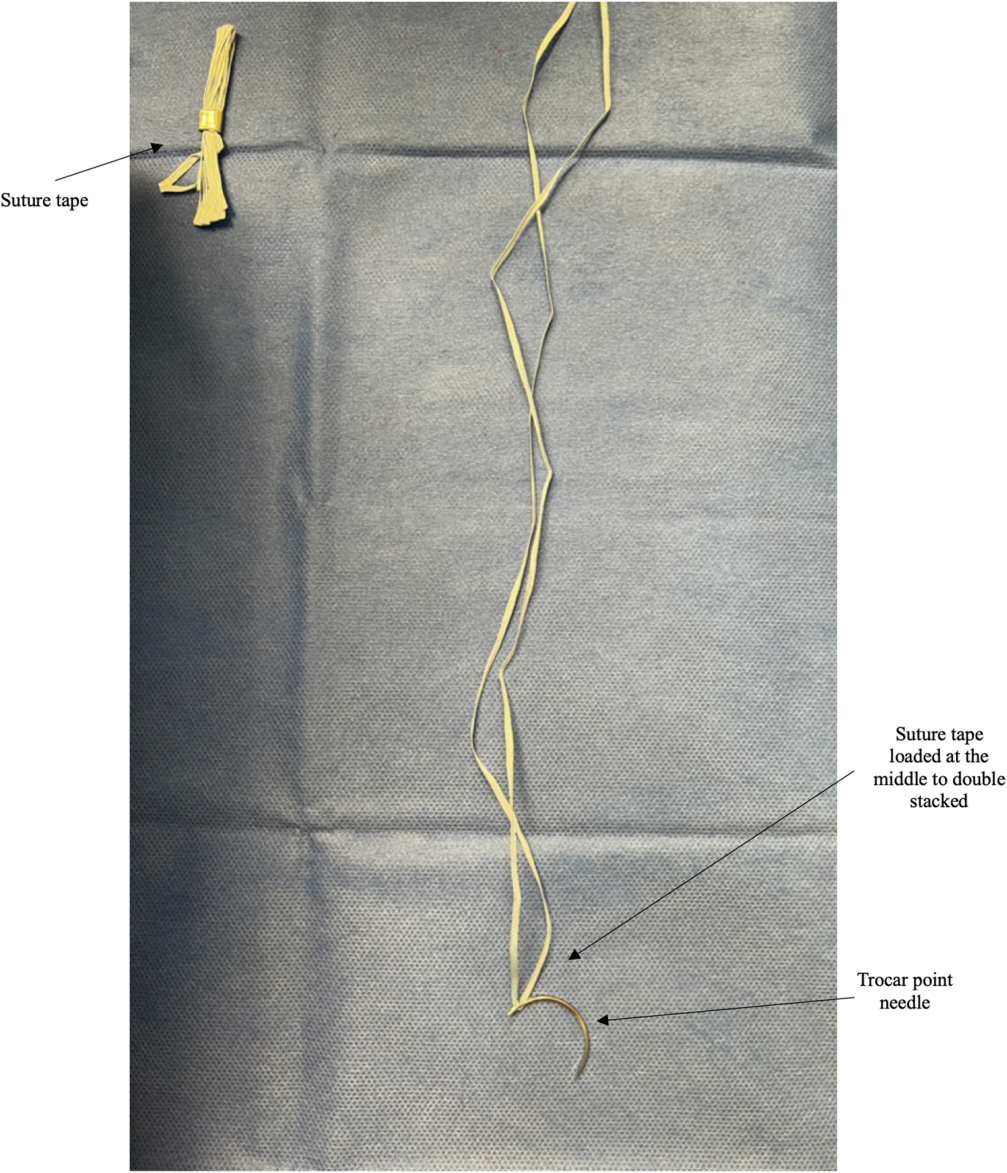
Top left shows a suture tape when removed from the manufacturer's package. The right shows the unwound suture tape loaded at the middle using a trocar point needle to provide a double-stacked suture tape unit.

Non-absorbable suture tapes are used instead of cerclage wire. The tapes distribute the forces across the soft tissue and maintain reduction. The suture tapes are double stacked by loading a trocar point needle at the midpoint (Figure 1).

**Figure 2:**
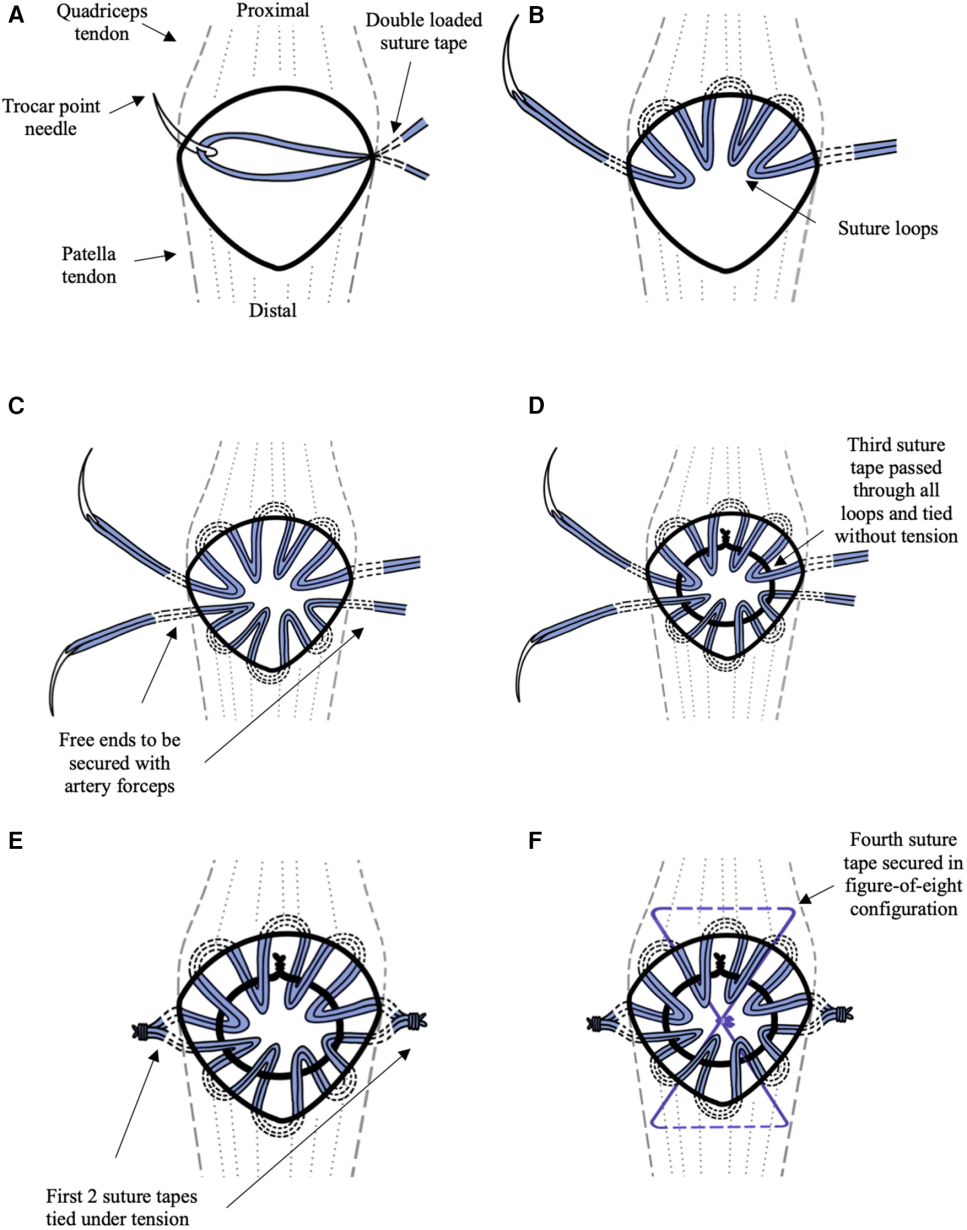
The illustrations **(A–F)** lay out the steps of the technique as explained in the Technique section. The first two double-stacked suture tapes are illustrated in blue. The third suture tape is illustrated in black. The fourth suture tape is illustrated in violet. **(A)** The double-stacked suture tape unit is advanced using trocar point needle into the retinaculum at the medial edge of the patella. **(B)** The suture tape unit is passed several times through the soft tissue around the edge of the superior half of the patella creating four loops. **(C)** Step 2b is repeated for the inferior half of the patella. **(D)** The third double-stacked suture tape is used to pass through all the suture loops and tie onto itself to form a circle at the anterior surface of the patella without tension. **(E)** The suture tapes are pulled uniformly thereby compressing the fragments together. **(F)** The fourth double-stacked suture tape is passed beneath the quadriceps tendon proximally and at patella tendon distally forming a figure-of-eight configuration tension band.

**Figure 3:**
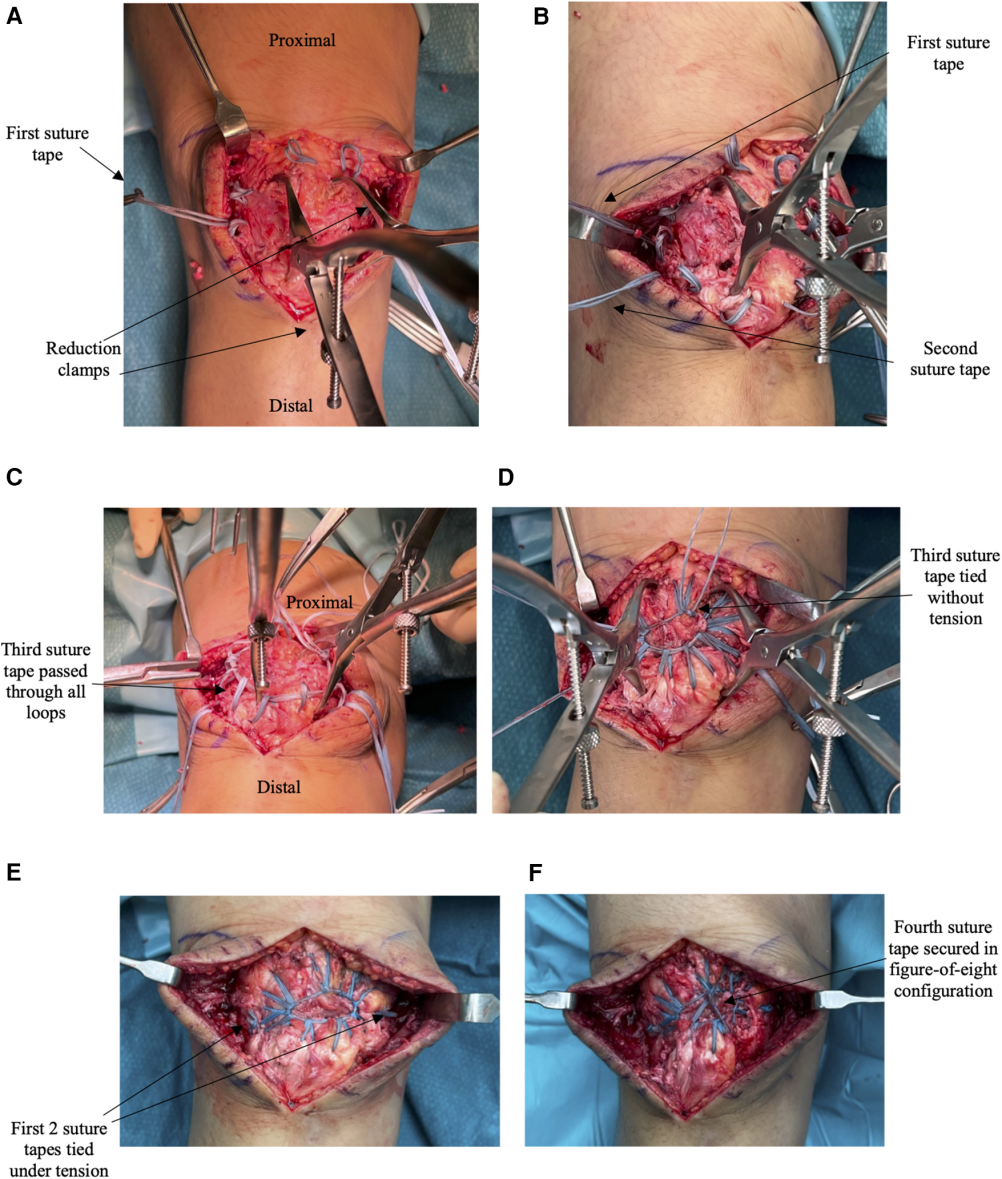
**(A–F)** lay out the intraoperative images showing the steps of fixation with the corresponding illustration in Figure 2 stated. **(A)** The patella fracture is reduced using two clamps. The first double-stacked suture is passed loosely creating loops at the superior half of the patella with the free ends at the medial and lateral edges of the patella. This corresponds to the illustration in Figure 2B. **(B)** The step is repeated with the second double-stacked suture tape passed through the inferior half of the patella. This corresponds to the illustration in Figure 2C. **(C)** The third double-stacked suture tape is passed through the first two suture tapes’ loops. **(D)** The third double-stacked suture tape ties onto itself to form a circle at the anterior surface of the patella. This corresponds to the illustration in Figure 2D. **(E)** The first two suture tapes are tied under tension with the excess free ends removed. This corresponds to the illustration in Figure 2E. (**F**) The final construct shows the fourth suture tape tied in the figure-of-eight configuration. This corresponds to the illustration in Figure 2F.

The fractured patellar is divided into proximal and distal halves by the fracture line. First, a suture tape unit is passed using the trocar point needle. This starts at the level of the retinaculum tear on one side of the patella and ends on the opposite side (Figure 2A). The tape is passed several times through the soft tissue with a minimum of three to four loops of suture tape created on one half of the patella (Figures 2B, 3A). The free ends of the tape on the medial and lateral aspect of the patella are temporarily secured with an artery forceps to prevent migration. Similar steps are taken to pass the suture tapes around the other half of the patella (Figures 2C,  3B). This creates multiple suture loops around the center of the patella with the ends of the suture tapes at either side of the patella.

A third double-stack suture tape unit is used to pass through all the suture loops (Figure 3C) and tied onto itself to form a circle at the anterior surface of the patella (Figures 2D, 3D). This loop is tied off without attempting to tighten the tape. The aim is to achieve a small center loop of the tape that is large enough to avoid inducing tension in the surrounding radially oriented tape loops.

The first two suture tapes are then tensioned uniformly to remove any slack in the suture tapes and thereby compressing the fracture fragments together (Figures 2E, 3E). The excess tape arms are then cut off using a scalpel. As an augmentation, a fourth double-stacked suture tape unit is used to create a tension band fixation by passing it beneath the quadriceps tendon proximally and patella tendon distally in a figure-of-eight configuration (Figures 2F, 3F).

Subsequent to fracture fixation, the stability of the fixation construct is checked through passive knee ranging. A final lateral radiograph of the knee is then taken to ensure satisfactory articular reduction and fixation. Post-operatively, the patients are allowed to bear weight as tolerated with the affected knee protected by a functional brace under the supervision of a physiotherapist.

## Expected outcome

One of the main benefits of this technique is that it is performed with the knee in full extension, which is advantageous particularly in complex fracture patterns. As the extensor mechanism relaxes in full extension, this negates the strong tensile forces of the quadriceps on the patella and eases reduction. This technique also allows reduction in extent of incision and soft-tissue dissection as parallel k-wires or screw insertion with traditional techniques is not required.

Suture tape is composed of ultra-high-molecular-weight polyethylene, which is highly biocompatible and inert. The low tissue may reactively reduce wound complications or infection rates. The softness and thin profile of the suture tapes confer much lower risk of implant prominence and soft-tissue irritation compared with metallic hardware. This in turn translates to reduction in the need for re-operations for removal of implants.

The double-stacked suture tape unit confers sufficient tensile strength when it is tied to itself; this way it has been shown to be superior to sutures in biomechanical study. The low memory and flexibility of the suture tape provides ease of handling compared with metallic wires. It can be more easily passed in close proximity to the circumference of the patella compared with a conventional wire and hence is able to provide strong circumferential compression. With the trocar point needle, the surgeon is provided with a method of fixation using familiar wrist motion of passing a suture in soft tissue.

This novel technique for patella fracture is simple to perform and does not require special instruments. This theoretically translates to shorter surgical time, with the mean operation time of 60 min in our center. The ease of achieving anatomical reduction and applying the suture tape also leads to decrease in usage of intraoperative imaging. The authors have managed to take less than five intraoperative images for each case.

**Figure 4:**
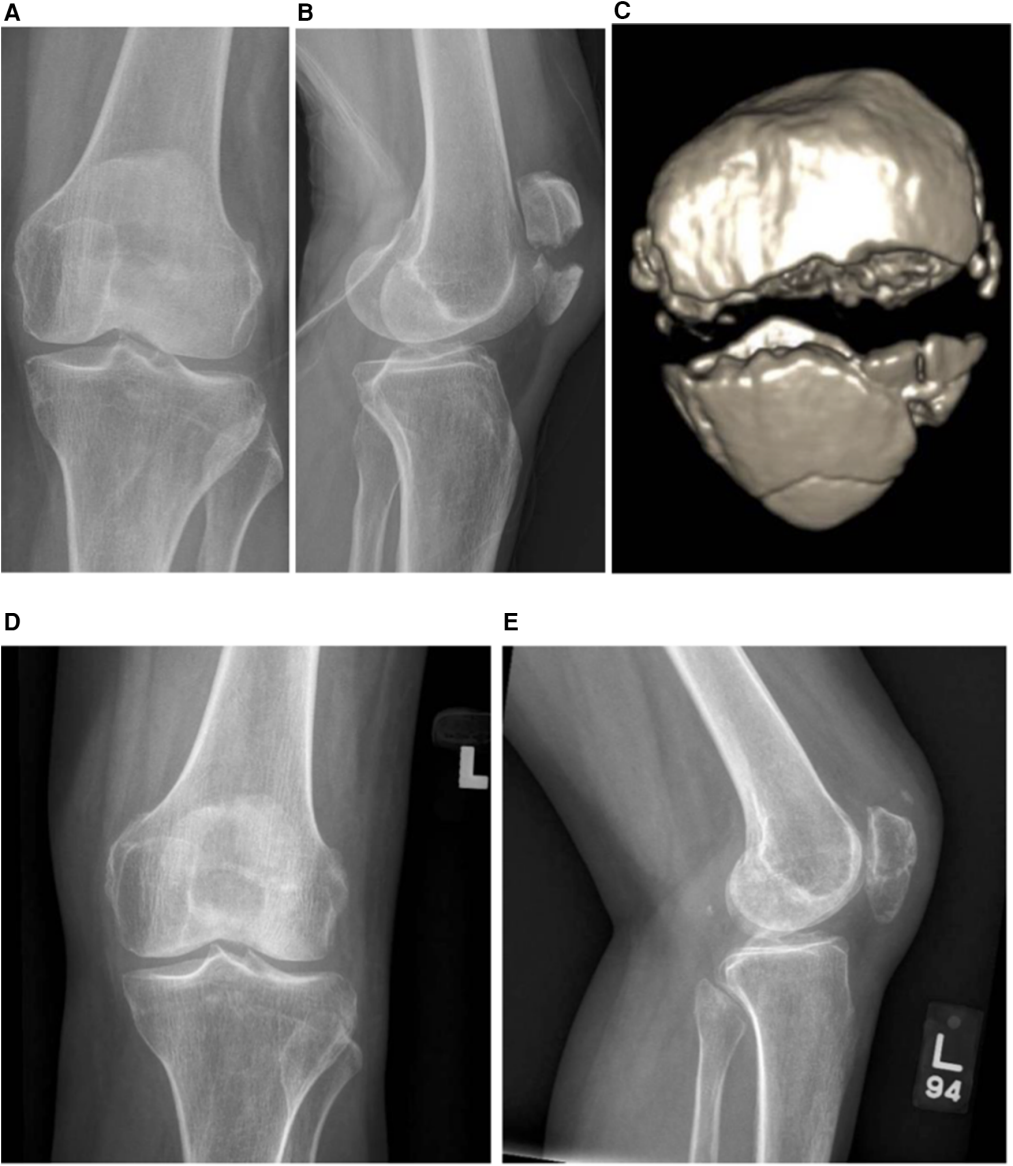
Case illustration of an AO34C2 patella fracture. **(A,B)** Anterior–posterior and lateral knee radiographs. **(C)** CT three-dimensional (3D) reconstruction of the patella fracture. **(D,E)** Post-operative knee radiographs at 3.5 months demonstrating satisfactory restoration of the articular surface and fracture union.

**Figure 5:**
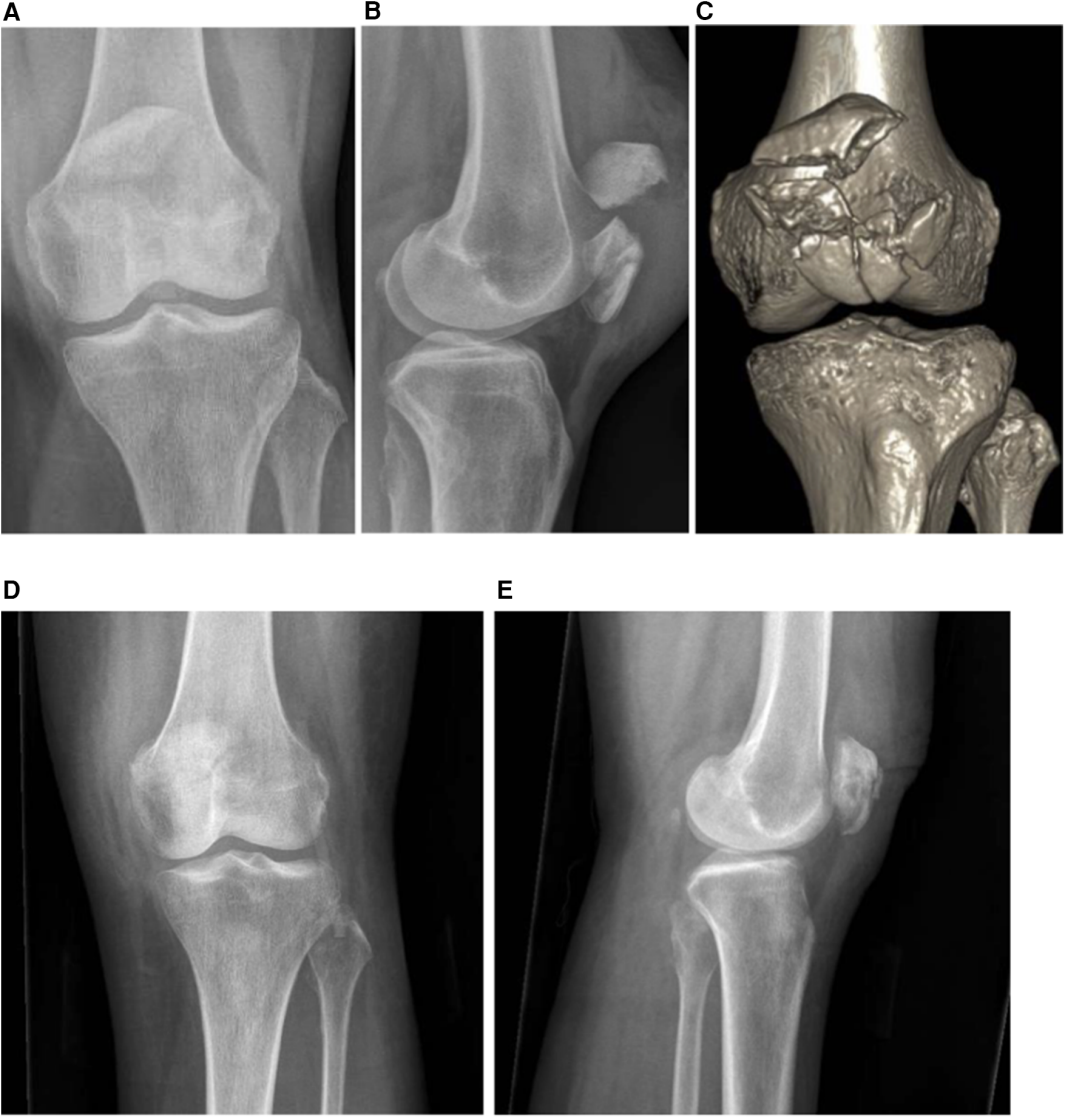
Case example of an AO34C3 patella fracture. **(A,B)** Anterior–posterior and lateral knee radiographs. **(C)** CT 3D reconstruction of the patella fracture. **(D,E)** Post-operative knee radiographs at the 3-month follow-up demonstrating good articular congruency attained.

The early results of two cases are reported in this paper. Case 1 is a 62-year-old lady who sustained a close transverse patella fracture and a split of the distal fragment (AO34C2) (Figure 4), while Case 2 is a 52-year-old lady who fell and landed on her knees (Figure 5) sustaining comminuted patella fracture (AO34C3). Both patients underwent the surgical fixation of the patella fracture using the proposed novel technique described in this paper. Union was achieved at the 3-month follow-up without fracture displacement. Both were able to return to the pre-operative range of motion of 0°–120° at 3 months as well. There were no reported complications such as loss of reduction or implant irritation necessitating revision surgery at the 1-year follow-up.

## Complications and pitfalls

The potential surgical complications of bleeding, nerve injury, surgical site infection, and post-operative stiffness would be expected to be similar to that of conventional methods of fixation, which are reported to be less than 3% (1). Non-union rates are expected to be low in patella fracture due to the rich blood supply conferred by the genicular anastomosis. Unfortunately, our small series prevents us from providing definitive data confirming the complication rate in comparison with conventional surgery.

This proposed surgical technique relies on the integrity of the surrounding soft tissue to hold the fracture fragments together as the suture tape is placed around the patella through the capsule. This technique is not suitable to use in fractures involving small superior or inferior pole fragments and quadriceps tendon or patella tendon tears/avulsions. In such cases it is not possible to achieve adequate soft-tissue fixation on a small bone fragment.

The initial two suture tapes are each placed around one half of the patella and achieve fixation on the capsular soft tissue posterior to the equator of the patella. The third suture tape is placed through the loops and tied to itself to form a suture ring. It is important to note that the third suture tape is not tightened. Next, the two circumferential suture tapes are tensioned and tied on either side of the patella. This creates a circumferential compression at the level of the articular surface. Failure to place the capsular stitches in a posterior/dorsal position may result in compression at the superficial patella and an unintended convex deformity.

Due to the novelty of the surgical technique we have yet to perform any biomechanical test or cadaveric study to evaluate the dynamic performance of the fixation. As with all pioneering techniques, the long-term performance and outcome remains unknown, consequently patients should be monitored and followed closely. The authors are conducting a cohort study with a bigger sample size and a longer follow-up period to validate the efficacy and safety of this proposed technique. A biomechanical study comparing our proposed technique with the traditional method of patella fixation is also underway.

## Conclusion

This preliminary technical paper offers a glimpse into the feasibility of using non-metal implants in fixing subcutaneous bone and challenges the status quo for fixing fractures with metals. The included cases have shown promising results as both patients managed to achieve union, satisfactory range of motion with no loss of fracture reduction, and no complication. We believe that more research and development is needed in the field of material science to explore non-metallic implant alternatives for fixation of fractures.

## Data Availability

The original contributions presented in the study are included in the paper/Supplementary Material, further inquiries can be directed to the corresponding author.
